# Serum Soluble Interleukin-2 Receptor is Increased in Malnourished and Immunosuppressed Patients With Gastric and Colorectal Cancer: Possible Influence of Myeloid-Derived Suppressor Cells

**DOI:** 10.4021/wjon548e

**Published:** 2012-08-26

**Authors:** Kenji Gonda, Masahiko Shibata, Tatsuo Shimura, Takeshi Machida, Satoshi Suzuki, Izumi Nakamura, Shinji Ohki, Kenichi Sakurai, Hitoshi Ohto, Ryouichi Tomita, Seiichi Takenoshita

**Affiliations:** a Department of Blood Transfusion and Transplantation Immunology, Fukushima Medical University, 1Hikarigaoka, Fukushima, Fukushima 960-1295, Japan; b Department of Organ Regulatory Surgery, Fukushima Medical University, 1Hikarigaoka, Fukushima, Fukushima 960-1295, Japan; c Department of Tumor and Host Bioscience, Fukushima Medical University, 1Hikarigaoka, Fukushima, Fukushima 960-1295, Japan; d Department of Immunology, Fukushima Medical University, 1Hikarigaoka, Fukushima, Fukushima 960-1295, Japan; e Department of Surgery, Nihon University School of Medicine, 30-1 Otaguchi-kamimachi, Itabashi, Tokyo 173-8610, Japan; f Department of Surgery, Nippon Dental University, 2-3-16Fujimi, Chiyoda, Tokyo 102-8158, Japan

**Keywords:** Gastric cancer, Colorectal cancer, sIL-2R, MDSC

## Abstract

**Background:**

Soluble IL-2 receptor (sIL-2R) is the circulating form of a membrane receptor localized on lymphoid and some tumor cells; its biological function is not completely understood.

**Methods:**

Serum levels of sIL-2R in blood samples taken from 51 cancer patients (21 gastric and 30 colorectal) and 18 healthy volunteers were measured and found to be significantly higher in the patients.

**Results:**

Concentrations were significantly inversely correlated to nutritional parameters, including total protein and short turnover proteins such as prealbumin, retinol binding protein and transferrin, as well as to the stimulation index, which is a classical parameter of cell-mediated immunity. Concentrations were significantly positively correlated to neutrophil count and inversely to lymphocyte count. Significantly elevated levels of circulating myeloid-derived suppressor cells (MDSCs) were found in patients; this was significantly correlated to sIL-2R levels.

**Conclusions:**

Increased production of sIL-2R correlated with systemic inflammation, nutritional impairment and inhibition of cell-mediated immunity, and thus may be involved in immunological mechanisms inducing cancer cachexia. The same factors also appear to relate closely to induction of MDSCs.

## Introduction

Cancer growth and development is associated with stimulation of the innate immune system, including enhanced expression of interleukin 2 receptor (IL-2R) in immune cells [1]. The high-affinity IL-2R consists of three chains: alpha (CD25), beta (CD122) and gamma (CD132). Beta and gamma chains are constitutively expressed on lymphocytes; both have long cytoplasmic domains that activate cytoplasmic proteins of the JAK-STAT pathway following binding of IL-2 to the trimeric receptor. The alpha chain is inducible and lacks a signaling function because of its short cytoplasmic domain. The main function of the alpha chain is to bind IL-2 and then promote optimal IL-2 signaling through the high-affinity receptor upon association with the beta and gamma chains [2]. In 1984, Rubin and coworkers reported the presence of soluble IL-2 receptors (sIL-2R) in cultured human T-cell leukemia virus 1 (HTLV 1)-positive T lymphocytes and in mitogen-stimulated peripheral blood mononuclear cells (PBMCs) [3]. A number of investigations have been conducted to gain better understanding of sIL-2R function, which remains somewhat unclear, but seems to reflect T-lymphocyte activation in many different diseases [4].

Many cancer immunotherapies developed in experimental animals have been tested in clinical trials. Although some have shown modest clinical effects, most have not [5, 6]. Recent studies have identified myeloid-origin cells that are potent suppressors of tumor immunity and therefore a significant impediment to cancer immunotherapy. Suppressive myeloid cells were described three decades ago in patients with cancer [7, 8], but their functional importance in the immune system has only recently been appreciated [9-12]. Indeed, accumulating evidence has now shown that myeloid-derived suppressor cells (MDSCs) contribute to the negative regulation of immune responses during cancer and other diseases. An accumulation of MDSCs was associated with decreased numbers of dendritic cells in the peripheral blood of patients with several types of cancer [12]. In contrast to the situation in mice, pathophysiological functions of MDSCs have not been as well clarified in patients. However, induction and expansion of the MDSC population is associated with autoimmunity and inflammation. Therefore, these suppressor cells may have a strong influence on development of inflammation and malignant disease.

Cachexia is characterized by weight loss, anorexia, anemia and hypoalbuminemia; its presence further increases cancer mortality. If not reversed, cachexia-associated derangements of homeostasis lead to immunological deficiencies, organ failure and multiple metabolic abnormalities. Cachexia is a complicated condition that has not yet been sufficiently characterized, but strong correlations have been reported with immune reactions in the cancer-bearing host [13]. The present study was designed to investigate the relationships, in patients with gastric and colorectal cancer, between the status of sIL-2R and markers of nutrition and inflammation, as well as levels of MDSCs.

## Methods

### Study subjects

Blood samples were collected from 51 patients, including 21 with gastric and 30 with colorectal cancer, and from 18 healthy volunteers with similar age and gender distributions. The gastric cancer patient group included 3 with stage I, 3 with stage II, 2 with stage III and 13 with stage IV disease, and of the 30 patients with colorectal cancer, there were 2 with stage I, 5 with stage II, 7 with stage III and 16 with stage IV disease. All enrolled patients had received surgery or chemotherapy in the department of Organ-Regulatory Surgery in Fukushima Medical University from January, 2011 to January, 2012 and were 45 - 89 years of age with histologically confirmed gastric or colorectal cancer. All patients were newly diagnosed and blood samples were collected before any treatment was initiated.

This study was approved by the ethics committee of Fukushima Medical University (2010-204) and written informed consent was obtained from all subjects enrolled in this study.

### Isolation of peripheral blood mononuclear cells (PBMCs)

PBMCs were isolated with Ficoll-Hypaque (Pharmacia-Biotech, Uppsala, Sweden), washed twice with RPMI1640 (Wako Pure Chemical Industries Ltd., Osaka, Japan) and stored in freezing medium (BLC-1, Juji-Field Co. Ltd, Tokyo, Japan) at -80 °C until use, all according to standard protocols.

### Serum levels of sIL-2R

Peripheral venous blood sera from all subjects were stored at -80 °C until use. Serum concentrations of sIL-2R were measured by enzyme-linked immunosorbent assay (ELISA) (R and D systems, Minneapolis, MN, USA) according to the manufacturer’s protocol.

### Flow cytometry

Cells were immunofluorescence labeled and analyzed by flow cytometry; labels used included fluorescent isothiocyanate (FITC), phycoerythrin (PE), and phycoerythrin cyanin 5.1 (PC5) (Beckman Coulter, Marceille, France). Antibodies were used at 10 µg/mL and 50 µg/mL and were diluted in PBS. Cells were incubated with antibodies for 20 min at 4 °C and washed with PBS. Antibodies used included FITC-conjugated CD14 (Abcam, Cambridge, UK), PE-conjugated CD11b (Beckman Coulter), and PC5-conjugated CD33 (Beckman Coulter). Data acquisition and analysis were performed on a FACSAriaII flow cytometer (BD Bioscience, Mountain View, CA, USA) using Flow Jo software (Tree star Inc. Ashland, OR, USA).

### Proliferation assay

Lymphocyte proliferation assays were carried out using PBMCs suspended in RPMI-1640 (Wako Pure Chemical Industries, Osaka, Japan) and 10% fetal calf serum (Sigma, St. Louis, MO, USA). Phytohemmaglutinin (PHA) mitogenesis was observed in PBMCs during 80 h of incubation with 10 µg/mL PHA at 37 °C in 5% CO_2_ with the addition of ^3^H-thymidine (Japan Radioisotope Association, Tokyo, Japan) for the last 8 h. Cells were harvested and ^3^H-thymidine incorporation was counted using a liquid scintillation counter (Perkin-Elmer Inc., Waltham, MA, USA); results were expressed as counts per minute (cpm). The stimulation index (SI) was obtained by calculating total CPM/control cpm (the control being PBMC incubated with no added PHA).

### Nutritional status assessment

Nutritional status was determined by measuring serum concentrations of total protein (Biuret method) and prealbumin (turbidimetric immunoassay), retinol binding protein (latex agglutination immunoassay), and transferrin (turbidimetric immunoassay), using standard clinical laboratory protocols as indicated.

### Statistical analysis

Differences between the groups were determined using the student’s t-test. Relationships between two variables were quantified by Spearman’s rank correlation coefficient. Significance was assumed at P < 0.05. Not all blood samples were of sufficient volume for all measurements.

## Results

### Serum sIL-2R levels

**Figure 1:**
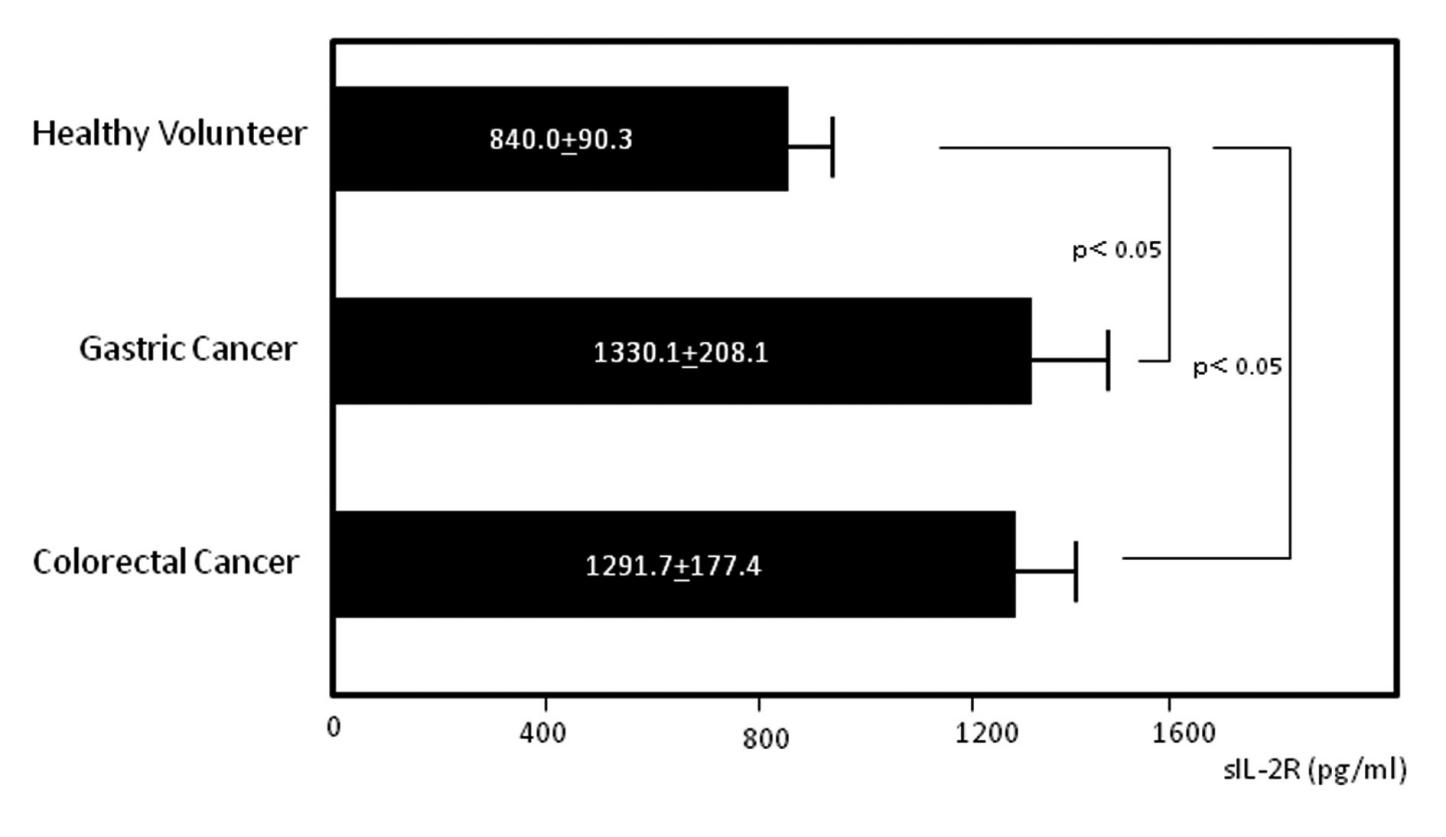
Serum levels of soluble interleukin-2 receptor (sIL-2R). Serum sIL-2R concentrations in gastric and colorectal cancer patients and healthy volunteers.

Serum concentrations of sIL-2R in gastric cancer patients, colorectal cancer patients and healthy volunteers were 1330.1 ± 208.1, 1291.7 ± 177.4, and 840.0 ± 90.2 pg/mL, respectively (Fig. 1). Levels were significantly higher in the gastric and colorectal cancer patients compared to healthy volunteers (P < 0.05 for both groups). No statistical differences were found among patients at different stages in either cancer type (data not shown).

### Flow cytometric analysis of MDSC

**Figure 2:**
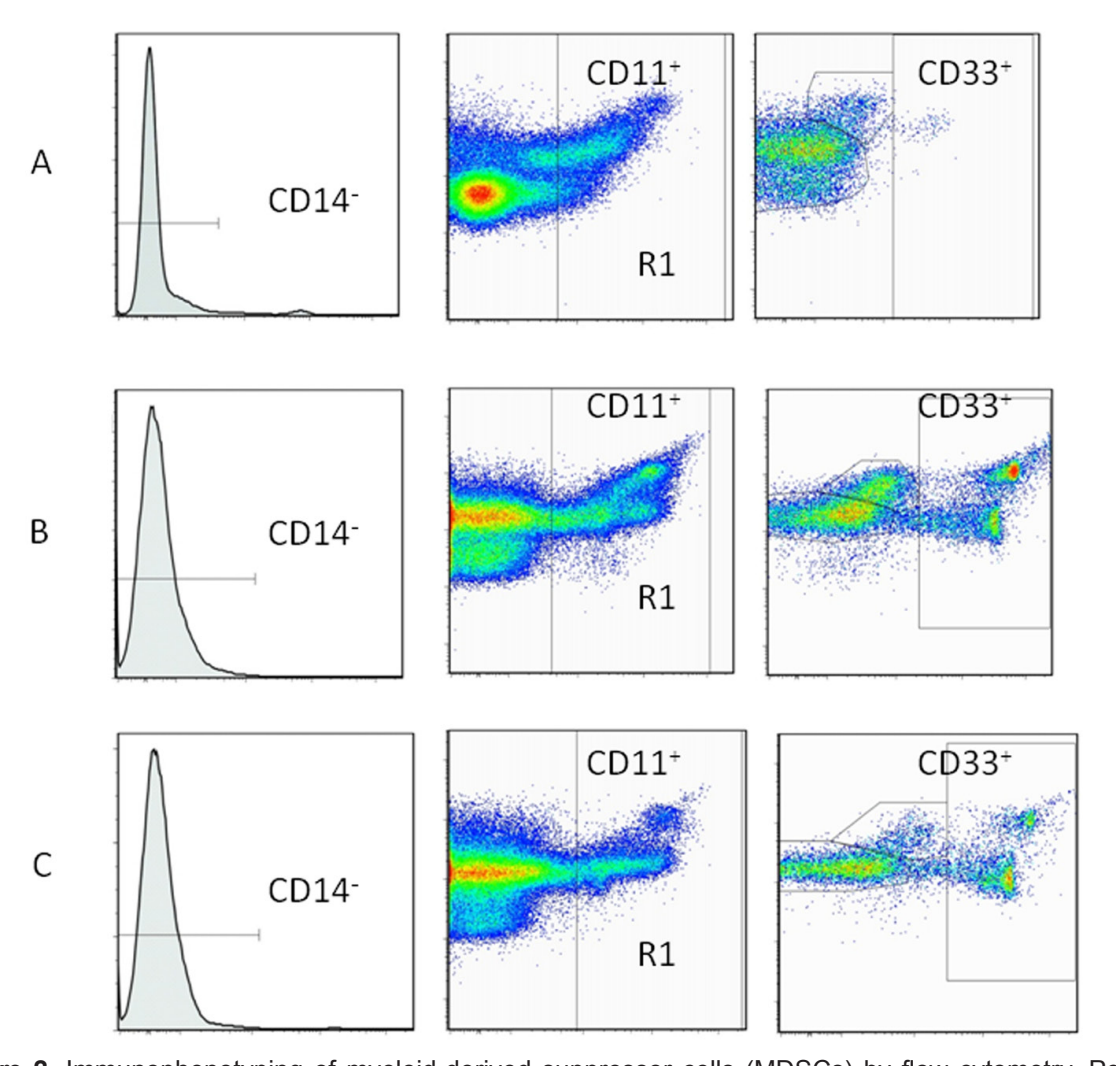
Immunophenotyping of myeloid-derived suppressor cells (MDSCs) by flow cytometry. Peripheral blood mononuclear cells (PBMCs) used for the flow cytometric analysis of MDSCs (CD11b+, CD14-, CD33+). A: healthy volunteers. B: gastric cancer patients. C: colorectal cancer patients.

**Figure 3:**
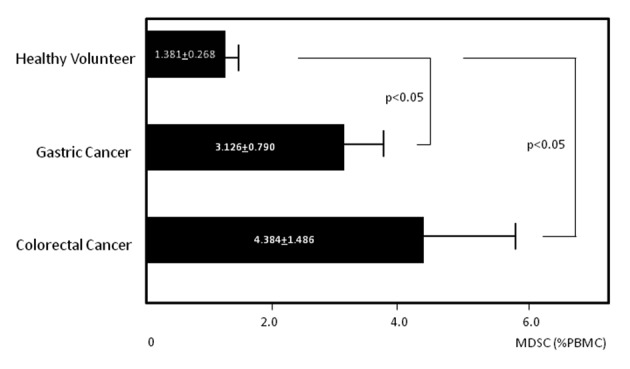
Circulating levels of myeloid-derived suppressor cells (MDSCs). Circulating levels of MDSCs in gastric and colorectal cancer patients and healthy volunteers.

MDSC levels were quantified by flow cytometry (Fig. 2). Circulating MDSC levels in gastric cancer patients, colorectal cancer patients and healthy volunteers were 3.126 ± 0.790, 4.384 ± 1.486, and 1.381 ± 0.268%, respectively. Levels in cancer patients were significantly elevated compared to those in healthy volunteers (P < 0.05 for both cancer types) (Fig. 3). Again, no statistical differences were found among patients at various stages in either cancer group (data not shown).

### Nutritional status correlations

**Figure 4:**
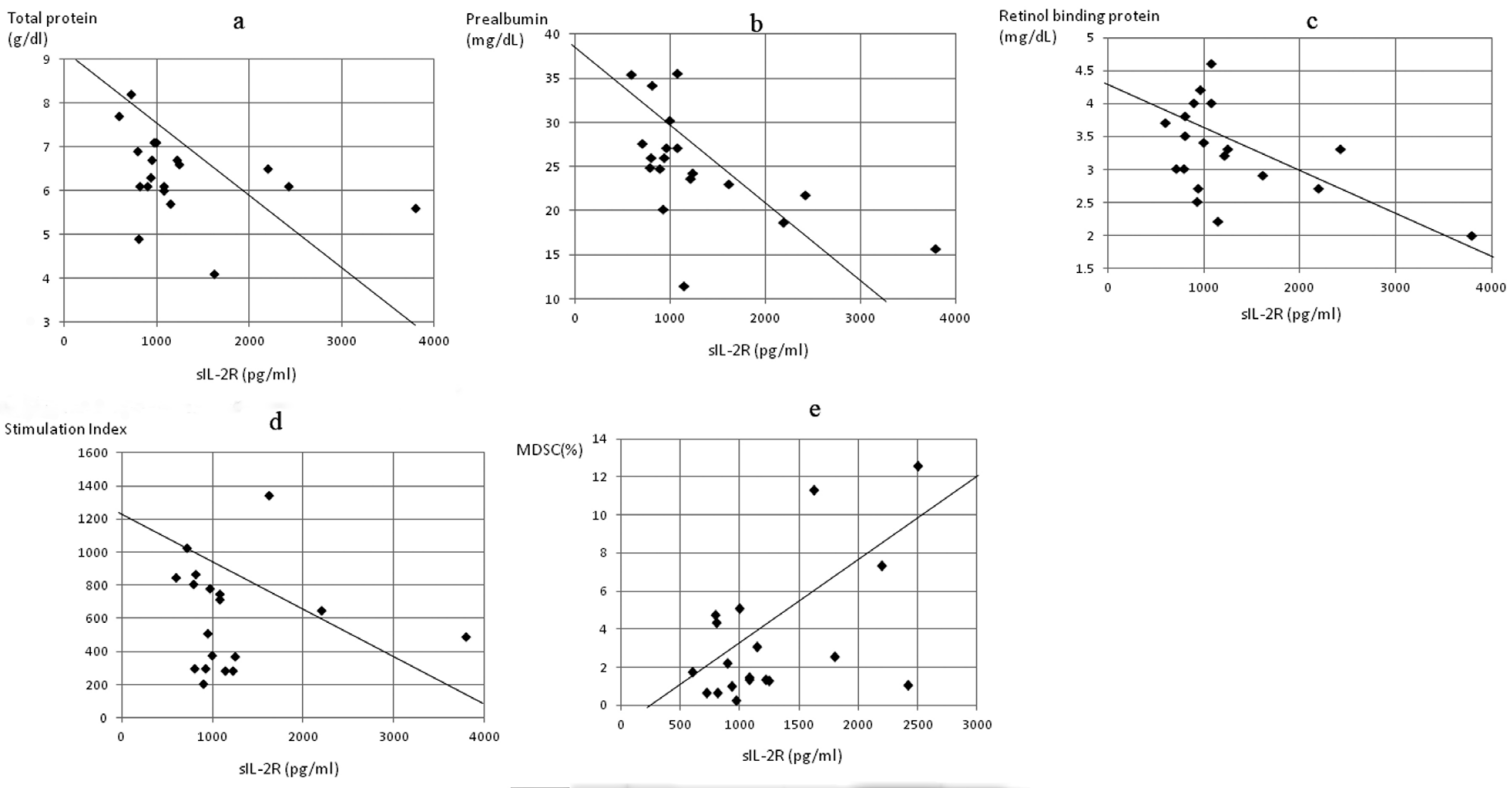
Correlation of serum concentrations of soluble interleukin-2 receptor (sIL-2R) to markers of nutrition, cell-mediated immunity and MDSC concentration in gastric cancer patients. The sIL-2R level was significantly inversely correlated to (a) total protein (P < 0.05, r = -0.413), (b) prealbumin (P < 0.0005, r = -0.784), (c) retinol binding protein (P < 0.05, r = -0.495), and (d) stimulation index (P < 0.05, r = -0.512), and positively correlated to (e) MDSC levels (P < 0.05, r = 0.482).

**Figure 5:**
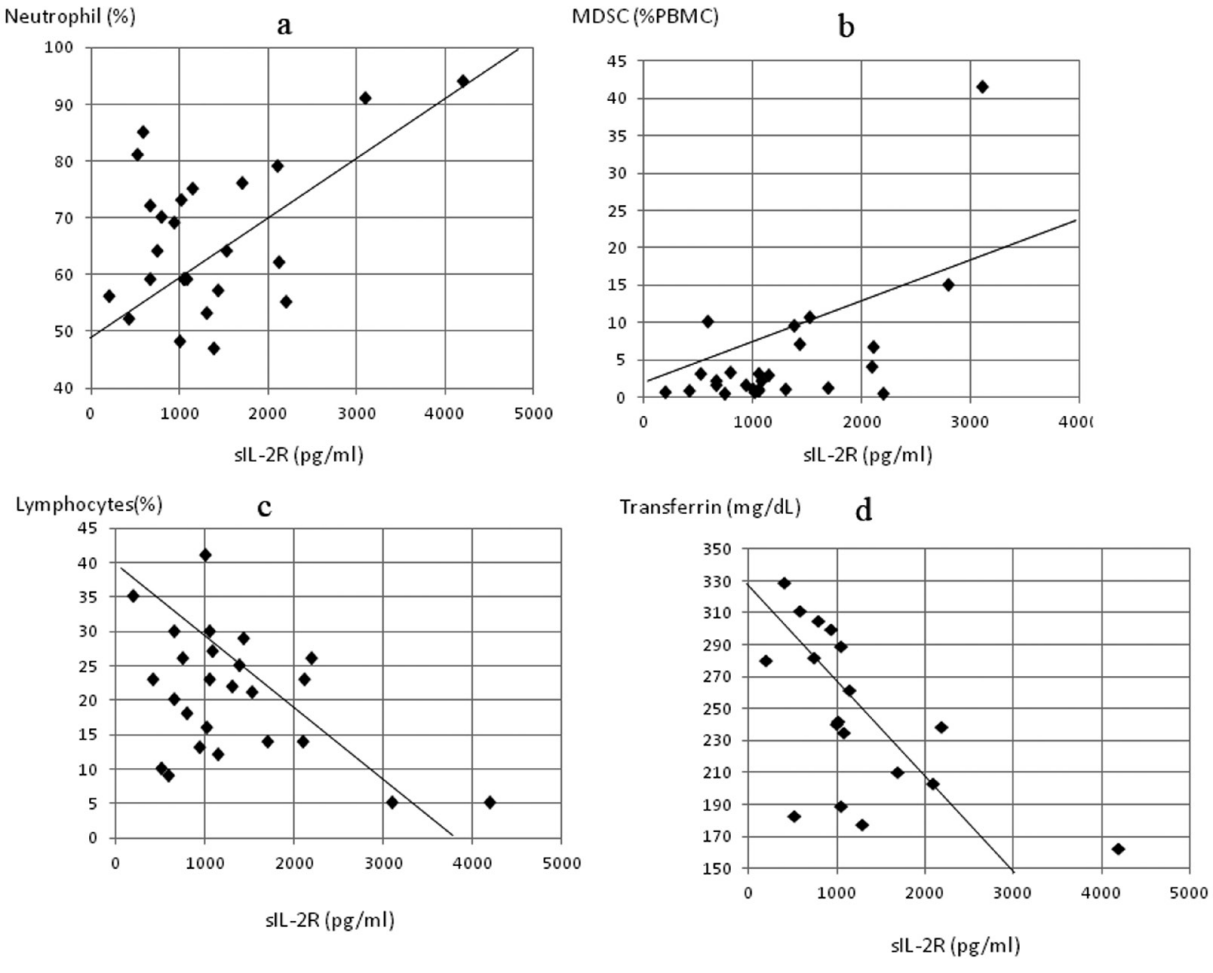
Correlation of serum concentrations of soluble interleukin-2 receptor (sIL-2R) to markers of inflammation, nutrition and MDSC concentration in colorectal cancer patients, sIL-2R significantly correlated to (a) neutrophil count (P < 0.01, r = 0.501) and (b) MDSC levels (P < 0.05, r = 0.453) and inversely correlated to (c) lymphocyte count (P < 0.05, r = -0.472) and (d) serum transferrin (P < 0.0005, r = -0.614).

In patients with gastric cancer, sIL-2R levels were significantly inversely correlated to levels of total protein (P < 0.05, r = -0.413) (Fig. 4a), prealbumin (P < 0.0005, r = -0.784) (Fig. 4b) and retinol binding protein (P < 0.05, r = -0.495) (Fig. 4c), and to the stimulation index (P < 0.05, r = -0.512) (Fig. 4d), while significant positive correlation was found with MDSC levels (P < 0.05, r = 0.482) (Fig. 4e). In patients with colorectal cancer, the sIL-2R level was significantly correlated with neutrophil count (P < 0.01, r = 0.501) (Fig. 5a) and MDSC level (P < 0.05, r = 0.453) (Fig. 5b) and inversely correlated to lymphocyte count (P < 0.05, r = -0.472) (Fig. 5c) and transferrin level (P < 0.0005, r = -0.614) (Fig. 5d).

## Discussion

Cancer growth and development is associated with stimulation of the immune system, including enhanced IL-2R expression in immune cells and subsequent shedding into the circulation. A number of studies have demonstrated critical connections among clinical symptoms of neoplasm, markers of inflammation, and sIL-2R levels in body fluids [1, 2, 14, 15]. This study demonstrated that serum levels of sIL-2R were significantly higher in patients with gastric and colorectal cancer and were significantly inversely correlated with several nutritional parameters and the stimulation index, a classical parameter of cell-mediated immunity. Serum levels of sIL-2R also were correlated positively with neutrophil count and inversely with lymphocyte count. Moreover, there were significantly elevated numbers of circulating MDSCs in patients that correlated with sIL-2R levels. The exact mechanisms involved in increased production of immature myeloid cells in cancer patients remain unclear. However, it is known that tumor cells may produce several growth factors and cytokines able to stimulate myelopoiesis [16-18]. In addition, vascular endothelial growth factor, produced by many tumors, can affect myelopoiesis. It is possible that increased production of these growth factors may affect normal cell differentiation, resulting in the accumulation of immature myeloid cells. MDSC numbers have been reported to decrease during certain cancer chemotherapy regimens [19], raising possibilities for future development of therapeutic modulation of these suppressive cells.

Malignant cells of human lymphoid tumors are thought to be the major source of circulating sIL-2R. However, it was demonstrated that some nonlymphoid cancer cells express surface IL-2 receptors [1, 2], while others do not. Malignant diseases have been found to be associated with impairment of T-cell-mediated immunity, and IL-2 and its membrane receptor were shown to be crucial to this process. As the finding in this study of inverse correlation to the stimulation index demonstrates, sIL-2R appears to be an inhibitory marker of cell-mediated immunity. Also shown was a correlation between sIL-2R levels and nutritional impairment as well as systemic inflammation. Increased neutrophils and decreased lymphocytes are sometimes seen in far-advanced cancer patients, and the neutrophil/lymphocyte ratio has been used as one of the easiest and most effective markers of chronic inflammation and the related immune suppression occurring in those patients [20]. The present data also suggest that sIL-2R is increased further in terminal stages when nutritional status is impaired, as evidenced by hypoproteinemia. Previously reported results indicated that suppression of cell-mediated immune reactions was closely related to nutritional status [13, 21], and that this seems to play a role in development of cancer cachexia.

### Conclusions

This study demonstrated that increased production of sIL-2R correlated with systemic inflammation, nutritional impairment and inhibition of cell-mediated immunity, and thus may be involved in an immunological mechanism for induction of cancer cachexia. The same factors also seemed to relate closely to induction of MDSC. Future studies are warranted to investigate possibilities for clinical control of immune suppression and chronic inflammation through modulation of IL-2 receptor expression or MDSC production using selective inhibition by molecular targeting or chemotherapy.
